# A mechanistic model of tau amyloid aggregation based on direct observation of oligomers

**DOI:** 10.1038/ncomms8025

**Published:** 2015-04-30

**Authors:** Sarah L. Shammas, Gonzalo A. Garcia, Satish Kumar, Magnus Kjaergaard, Mathew H. Horrocks, Nadia Shivji, Eva Mandelkow, Tuomas P.J. Knowles, Eckhard Mandelkow, David Klenerman

**Affiliations:** 1 Department of Chemistry, University of Cambridge, Lensfield Road, Cambridge CB2 1EW, UK; 2 DZNE, German Center for Neurodegenerative Diseases, Ludwig-Erhard-Allee 2, 53175 Bonn, Germany; 3 CAESAR Research Center, Ludwig-Erhard-Allee 2, 53175 Bonn, Germany; 4 Max-Planck-Institute for Metabolism Research, Hamburg Outstation, c/o DESY, Notkestrasse 85, 22607 Hamburg, Germany

## Abstract

Protein aggregation plays a key role in neurodegenerative disease, giving rise to small oligomers that may become cytotoxic to cells. The fundamental microscopic reactions taking place during aggregation, and their rate constants, have been difficult to determine due to lack of suitable methods to identify and follow the low concentration of oligomers over time. Here we use single-molecule fluorescence to study the aggregation of the repeat domain of tau (K18), and two mutant forms linked with familial frontotemporal dementia, the deletion mutant ΔK280 and the point mutant P301L. Our kinetic analysis reveals that aggregation proceeds via monomeric assembly into small oligomers, and a subsequent slow structural conversion step before fibril formation. Using this approach, we have been able to quantitatively determine how these mutations alter the aggregation energy landscape.

## Abstract (web summary)

Alzheimer's disease is characterized by the aggregation of the Abeta peptide and the tau protein. Here the authors track the formation of wild-type or mutant tau oligomers through to large aggregates and gain insights into the molecular basis of how tau mutations cause disease by altering the aggregation pathway.

## Graphical abstract

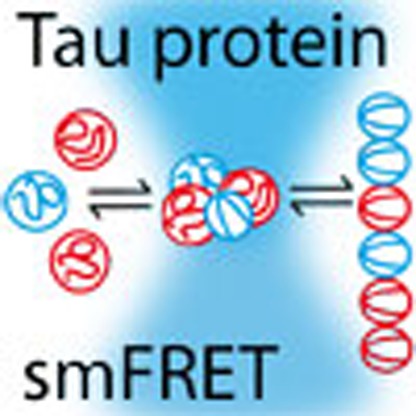

The spontaneous aggregation of proteins to form larger structures is a widespread and fundamental process in both normal and aberrant biology. Formation of large insoluble fibrils, often via self-assembly of soluble protein into oligomeric structures, is key to the pathology of several well-known human conditions such as Parkinson's disease^1^, prion disorders and Alzheimer's disease^2^. Alzheimer's disease is characterized by the deposition of two distinct types of aggregates—extracellular plaques composed of Aβ peptides (cleavage products of the transmembrane protein APP) and intracellular neurofibrillary tangles composed of hyperphosphorylated tau protein. Tau is a microtubule-binding protein that can aggregate into filaments, which are amyloid in nature (based on cross-β structure) and are the major constituents of neurofibrillary tangles in the neurons of Alzheimer-diseased brains^3^.

Tau proteins are broadly speaking divided into two domains—a carboxyl-terminal microtubule-binding domain and an amino-terminal projection domain^4^. The microtubule-binding domain is positively charged overall, assisting its interaction with the negatively charged surfaces of microtubules, and contains three or four similar, but not identical, repeat regions of 31 or 32 amino acids^5^ (Fig. 1). Tau is rich in polar amino acids, which renders it a highly soluble protein with little secondary structure^6^ even once bound to the microtubule^7^. At first glance, it is therefore a surprise that this protein assembles into amyloid structures. However, hexapeptide motifs, which are present in two of the four repeat regions of the microtubule-binding domain, have a high β-sheet-forming propensity^8^. These have been demonstrated to be fundamental to the aggregation process, and form the core of the filaments^8^,^9^. Aggregation of full-length tau *in vitro* is indeed generally slow because of the charged, highly soluble nature of the protein^10^. *In vitro* aggregation studies have therefore tended to focus on constructs formed from the aggregation-prone repeat domain of the microtubule-binding domain (K18 and K19), which aggregate faster without the presence of the flanking regions^11^. In addition, polyanion cofactors such as heparin and RNA have been found to accelerate aggregation, presumably by interacting with tau and compensating for the positive charges of the tau molecules, creating less unfavorable long-range electrostatic forces between tau molecules, and increasing the local tau concentration^6^,^12^,^13^,^14^. Heparin has traditionally been used to initiate aggregation within *in vitro* tau aggregation studies^14^.

Until relatively recently, only average populations of fibrils were readily accessible in both *in vivo* and *in vitro* experiments, with transient oligomer populations proving much more difficult to detect^15^. To date, most theoretical modelling and understanding has thus focused on describing fibril formation, leading to some successful analytical treatments^16^,^17^. However, recent developments in single-molecule fluorescence techniques have led to significant advances in our ability to also detect the formation of lowly populated oligomeric species. New theoretical models are now desired that are capable of fully describing these better-characterized aggregation kinetics, and quantifying them. Although early single-molecule observations of synuclein aggregation were described with a simple conversion model^18^, an explicit kinetic treatment providing rate constants for all microscopic steps in the entire aggregation reaction has not been achieved to date. In this work, we have applied kinetic analysis to single-molecule fluorescence measurements to study the aggregation of tau protein and determine the aggregation pathway and the number and size of tau oligomers formed during this process. This approach allows the detailed analysis and quantification of many aspects of aggregating systems that may be difficult to directly measure experimentally.

## Results

### Aggregation of K18 tau into paired helical filaments

**Figure 1:**
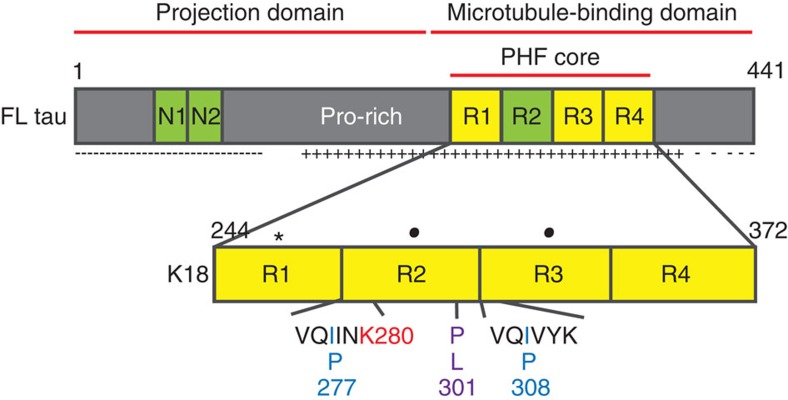
Cartoon of the full-length tau sequence, showing the major regions and location of the K18 sequence. Tau is alternatively spliced *in vivo*, with two possible inserts near the N terminus (N1 and N2) and one in the carboxyl-terminal half (R2). Repeats 1–4 (R1—R4), each 31 or 32 residues, represent the core of the filaments. The amino-terminal half of tau is termed ‘projection domain' because it does not bind to the microtubule wall. The point deletion ΔK280 (indicated in red) located in one of the two hexapeptide motifs responsible for aggregation of tau into filaments^43^, and the substitution P301L (indicated in purple) that occur in FTD strongly enhance the β-sheet-forming propensity. The anti-aggregant PP mutant contains two substituted proline residues at positions I277 and I308 (indicated in blue). The K18 construct^11^, consisting of the four repeat regions, has been adapted for this work through three mutations—C291A and C322A (black dots) and I260C (black star) to allow specific fluorophore Alexa labelling at position 260 using maleimide chemistry.

**Figure 2:**
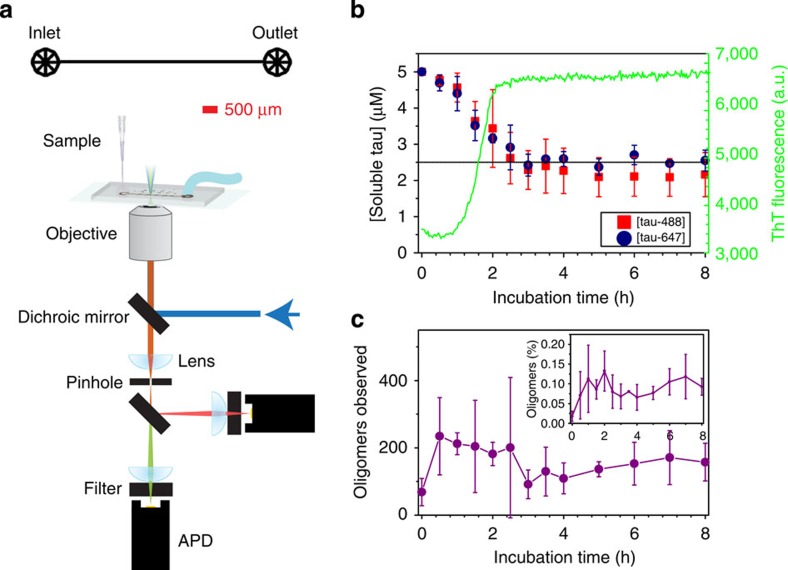
Aggregation of K18 under standard fibril-forming conditions followed using single-molecule FRET. (**a**) Diagram of microfluidic channel (upper) and single-molecule FRET set-up (lower) used in this work. (**b**) Bulk aggregation kinetics for the filament formation of K18 (left-hand axis). Soluble tau concentrations of A488-K18 and A647-K18 during the experiment. The dotted line represents the remaining soluble concentration of unlabelled K18 after 24 h when it is incubated under the same conditions (right-hand axis, green line). Formation of filaments by A647-K18 monitored by ThT fluorescence. (**c**) Number of detected oligomers throughout the aggregation. The inset is the fraction of soluble tau in oligomers, according to burst rates. Aliquots of reaction mixture were diluted rapidly and subjected to single-molecule FRET analysis. In **b**,**c**, each point represents the average of —three to five individual measurements, and the error bars the s.d.

We have performed single-molecule fluorescence studies of AlexaFluor-488 and AlexaFluor-647 labeled tau proteins (K18 construct) during their aggregation into filaments. This construct contains the four repeated sequences in the microtubule-binding domain that form the core of filaments and includes the two hexapeptide motifs in repeats R2 and R3, which nucleate aggregation^3^. We then compared its aggregation behaviour with that of a single point deletion mutant (ΔK280), one of the tau mutations found in frontotemporal dementia and parkinsonism linked to chromosome 17 (FTDP-17)^19^,^20^, and a single point mutation mutant (P301L) that is the most common mutation associated with FTDP-17 (ref. 21). Fluorophore-labelled tau constructs based on the K18 tau construct were created (Fig. 1)^10^,^11^. Our results from bulk studies indicate that the presence of the Alexa label at residue 260 (outside the filament core) does not significantly affect the aggregation process; filaments formed were visibly similar by transmission electron microscopy (Supplementary Fig. 1) and aggregation rates were similar (Supplementary Fig. 2). We first measured the overall progression of the association reaction using ThT fluorescence and soluble protein concentrations. Under these conditions, filament formation is ∼90% complete within 3 h (Fig. 2b).

**Figure 3:**
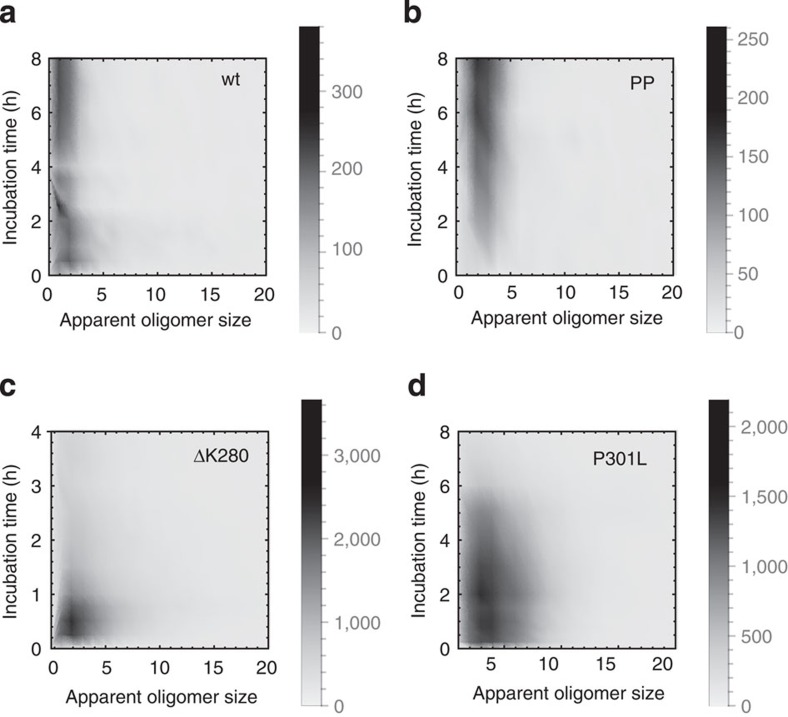
Number density plots showing the evolution of the apparent oligomer size of A488/A647-K18 and mutants at different incubation times. Oligomers remain small throughout the reaction, with no observed especially stable sizes. Numbers reported are the total of each oligomer size recorded during the —three to five replicate experiments.

A single-molecule fluorescence resonance energy transfer (FRET) approach was then used to observe any oligomeric species^18^. An equimolar mixture of A488-K18 and A647-K18 was incubated under filament-forming conditions. At various points throughout incubation, aliquots were removed and diluted to concentrations suitable for single-molecule fluorescence analysis. These solutions were then pumped under continuous flow through a microfluidic channel mounted on a microscope slide^22^ (Fig. 2). A blue (488 nm) laser beam was focused to a diffraction-limited confocal spot within the centre of the microfluidic channel, leading to the excitation of A488-K18. Under these conditions, A647-K18 monomers were undetectable, whereas A488-labelled monomers resulted in a single fluorescent burst in the blue channel. Oligomers containing both the labels were detected as bursts in the red channel, since any A647-K18 in close proximity to A488-K18 can be indirectly excited via FRET. We used this approach to quantify the proportion of the soluble (monomeric and oligomeric) material that was oligomeric. The vast majority of species observed were monomeric throughout the reaction. However, after only 0.5 h, where ∼95% of tau remained soluble, there was an increase in the number of oligomers (Fig. 2c), representing ∼0.1 % of the detected soluble tau species by number (Fig. 2c, inset). Most of the oligomers were only slightly brighter than the average monomer, and thus presumably relatively small. We estimated their apparent size by comparing the intensity of the oligomer fluorescence with that of a monomer, making the assumption that there was no significant quenching in the oligomeric species (see Methods)^18^. Pooling of all the data revealed that these small oligomers are most highly populated during the timeframe over which the filaments are formed and extended (Fig. 3, Supplementary Fig. 3). After ∼3 h, the majority of filament formation had taken place, and the oligomer concentration appeared stable.

**Figure 4:**
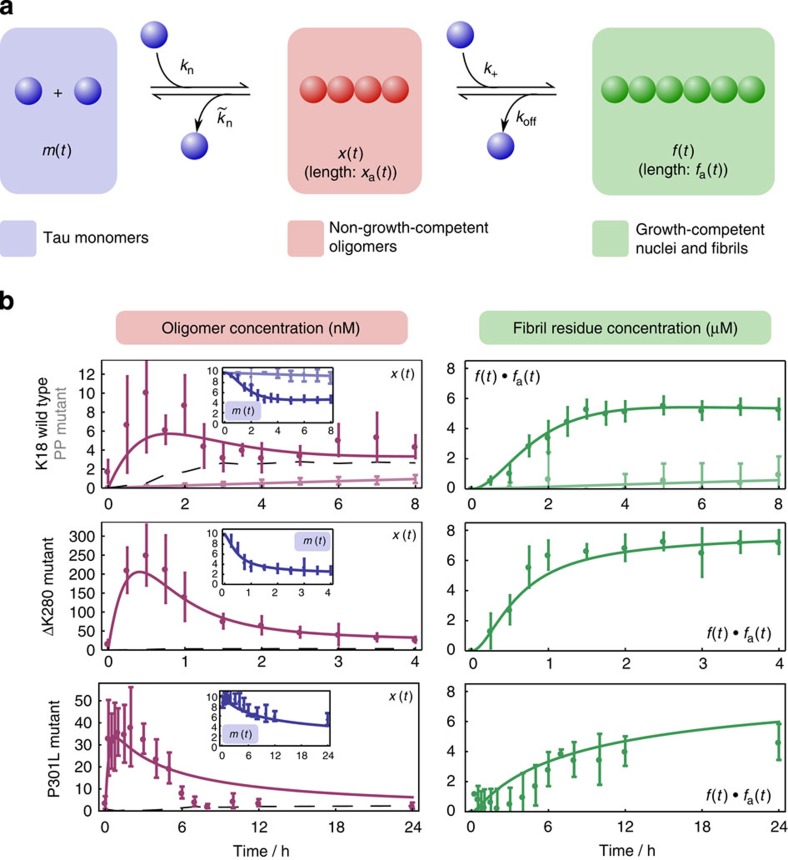
Nucleation–conversion–polymerization description of tau aggregation. (**a**) Coarse-grained on-pathway conversion model obtained from theoretical considerations (Supplementary Material). Initial formation of non-growth-competent oligomeric species *x*(*t*), of average length *x*_a_(*t*), occurs via a reaction of order *n*_c_ in monomer concentration *m*(*t*). Formation of fibrils *f*(*t*), of average length *f*_a_(*t*), then proceeds via addition of monomer units in a reaction of first order in *m*(*t*). Rate constants (*k*) for these processes and their corresponding reverse reactions are labelled. (**b**) Oligomeric and (deduced) fibrillar concentrations during the aggregation of K18, K18-PP, K18-ΔK280 and K18-P301L tau. Data represent the average of —three to five independent experiments, and error bars indicate the s.d. of these. Best fits for coarse-grained nucleation–conversion–polymerization description for K18, K18-ΔK280 and K18-P301L are shown as solid lines. The dotted black curves indicate upper bounds for *x*(*t*) predicted from the simpler inadequate nucleation–polymerization model (see Methods).

Observation of such a lowly populated species, which would certainly not be detected using traditional ensemble based methods, demonstrates the remarkable sensitivity of the technique for the early stages of the aggregation process. However, since the overall proportion of soluble tau in oligomers was so low, we took steps to ensure that we were detecting true oligomers in our experiments by examining an ‘anti-aggregant' mutant form of tau as a negative control. The K18-PP mutant has two proline residues introduced by substitution (I277P and I308P) to reduce its β-sheet-forming propensity and is reported in the literature to prevent tau aggregation^23^. Immediately following initiation of the reaction, the concentration of oligomers detected was around just 0.1 nM, as compared with typical oligomer concentrations of 5 nM detected for wild-type K18, confirming that we are observing true oligomers in our experiments with wild-type tau (Fig. 4). Surprisingly, on incubation under our standard conditions, the number of oligomers detected increased steadily and significantly throughout the monitoring period of 8 h (Supplementary Fig. 4, *P*<0.0001), though at a much slower rate than that observed for wild-type tau, and without any apparent formation of filaments. Since we have not observed any filament formation, we do not know if these oligomers are of the same kind as those seen for K18-wt, or indeed if they are on- or off-pathway; however, we note that they share a common size distribution with K18-wt (Fig. 3).

### Disaggregation of K18 tau paired helical filaments

**Figure 5:**
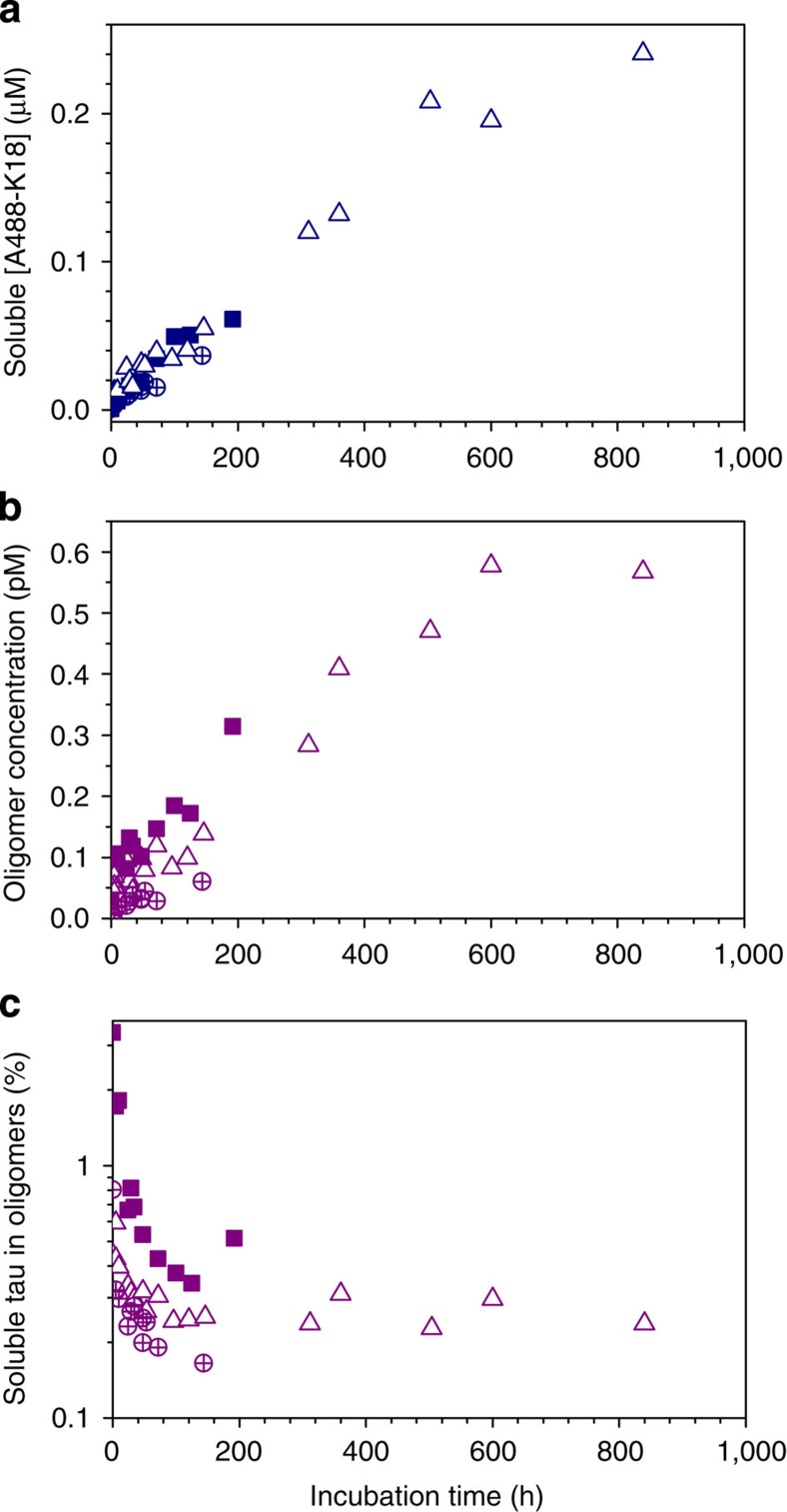
Fibrils formed from A488-K18 and A647- K18 tau disaggregate slowly into oligomers and monomers. (**a**) Soluble concentrations of A488-K18 were estimated using the burst rate in the donor channel from smFRET measurements. (**b**) The proportion of events due to oligomeric K18 decreases with incubation time, although the estimated total number of oligomers within the solution increased. (**c**) Three independent experiments were performed (open, filled and crossed symbols), the initial filament mass was similar but not identical in each.

We also investigated the dissociation process of the labelled filaments formed under our standard aggregation conditions to demonstrate that the aggregation reaction was reversible. Filaments of A488/A647- K18 were collected by centrifugation, and fresh buffer added on top to initiate dissociation. The concentration of soluble tau increased with incubation time as the equilibrium between fibrillar and soluble tau was re-established. Immediately following dilution of the sample, a relatively large proportion (0.6–3 %) of the soluble tau was oligomeric (as compared with 0.1 % observed during aggregation); however, this proportion decreased rapidly during the first 24 h after dilution as the monomeric concentration increased, despite the overall oligomeric concentration increasing steadily over 1 month (Fig. 5). The oligomers observed in this experiment were nearly all small, consistent with the species being populated towards the end of the aggregation experiments.

### Aggregation of two aggregation-prone K18 tau mutants

Having established a methodology, similar aggregation experiments were performed with the aggregation-prone deletion mutant of the K18 construct, K18-ΔK280. The aggregation reaction took place faster under the same conditions as K18 (Fig. 4a), as has previously been reported^20^, being largely complete within 1–2 h, so we changed the separation of the data points accordingly. Again, the majority of soluble species were monomeric (Fig. 4). The total number of detected oligomers peaked at 0.5 h, during the filament growth phase, and thereafter decreased, but remained higher than the number observed for K18-wt, even towards the end of the reaction. At peak, there were around 50 times more oligomers observed than for K18, where ∼4 % of the species detected were oligomeric (Fig. 4b). Owing to their larger population, we are better able to characterize the oligomers for this version of K18 tau, as demonstrated by the contour plots of the number distribution for K18-ΔK280 (Supplementary Fig. 3b). Similar to K18-wt, the vast majority of the observed oligomers remain small throughout the reaction (Fig. 3). We did not observe any peaks in the intensity distribution that would have suggested especially stable oligomer sizes; however, a small concentration of brighter species appear as the reaction proceeds and fibrils are created (Supplementary Fig. 3b). This could represent an increase in the average size of the oligomers or fibrils passing through the edges of the confocal spot, but represents a minority of the detected events.

Further aggregation experiments were then conducted with another mutated version of K18 associated with FTDP-17, K18-P301L. Oligomer concentrations reached a rapid peak ∼0.5 h after initiation of around—three to four times that observed for K18-wt, remaining high for some time before decreasing slowly as fibrils were formed (Fig. 4b). The oligomer size distribution is very consistent over the first few hours when few fibrils are present in the reaction mixture and oligomer concentrations are high, and strongly resembles those from both K18-wt and K18-ΔK280 (Fig. 3).

The larger concentrations of oligomers formed by the K18-ΔK280 mutant also allowed us to assess their stability on dilution. Oligomer-‘rich' samples were prepared by incubating K18-ΔK280 for 30 min under our standard conditions, and changes in the oligomer concentration following dilution were then monitored through the oligomer burst rate. The results of this experiment showed that some oligomers were dissociating throughout our typical measurement times, but that our estimated concentrations were underestimated by at most a factor of two (Supplementary Fig. 5).

Since earlier publications have reported on the toxic nature of small β-sheet-rich tau oligomers to the neuronal cell line SH-SY5Y^24^,^25^,^26^, we tested our samples of oligomer-containing K18-ΔK280 for such toxicity using a similar approach. Application of oligomers to the cell samples resulted in no detectable toxicity (Supplementary Fig. 6) even though we found the oligomers to be stable in cell culture medium throughout the incubation times used (Supplementary Fig. 7), possibly due to the tau in our experiments not being full length.

Oligomeric species are observed for all K18 tau types, and their formation broadly precedes fibril formation in aggregation reactions, with oligomer populations sequentially rising and falling before bulk depletion of monomer. Therefore, a key mechanistic question asks how the different timescales and amplitudes of the processes are related. A simple nucleation–polymerization model^16^,^17^,^27^,^28^, treating oligomers as small fibrils, cannot describe the observed oligomer population amplitudes while permitting the observed formation of long fibrils (Supplementary Fig. 8 and Online Methods). However, if oligomeric species are considered to be structurally distinct from small fibrils, the necessary introduction of an on-pathway conversion reaction successfully describes the reaction kinetics, for both the K18 construct and the ΔK280 and P301L mutants (Fig. 4).

Qualitative comparison of observations for K18 and its mutant versions highlights major differences between their kinetics. Oligomers are more highly populated for both (disease-associated) mutants, K18-ΔK280 and P301L. The overall timescales for the reactions are also different, for both the emergence of oligomers and filament accumulation. In the case of K18-ΔK280, the reaction reaches completion after ∼2 h compared with 4 h for K18, whereas the ‘anti-aggregant' PP mutant, displays no noticeable accumulation of fibrils within 8 h. It is also of note that although oligomers may have been observed for the ‘anti-aggregant' PP mutant, the oligomer concentrations have still not reached their peak within 8 h, suggesting that the nucleation rate constant is much lower for this mutant. In contrast, peak oligomer concentrations are reached rapidly for P301L and K18-ΔK280, suggesting that nucleation is significantly more favourable for these mutant tau species in comparison with wild type. We sought to describe these results in a more quantifiable fashion. Detailed quantification of these differences is made possible by numerically fitting our simple conversion model where monomers associate to form oligomers (of the apparent size observed in our experiments) in a heparin-dependent manner, and are subsequently converted into fibrillar species (Fig. 4a). Fitting to each of the data sets allowed the extraction of all relevant rate constants (Fig. 4b, Supplementary Table 1), and subsequently the changes in the activation free energy introduced on mutation for each reaction step, as described in Methods and summarized in Table 1. Since we had observed that there was some dissociation of oligomers during our measurements, we established that the estimated activation free energy changes were insensitive to the exact oligomer concentrations, that is, values did not change within error when all oligomer concentrations were doubled (see Methods). Although several mutation effects are apparent, the most striking one is the large increase in the initial nucleation rate on ΔK280 mutation. This effect primarily explains the higher number of oligomers observed for K18-ΔK280 aggregation, and is likely to reflect reduced electrostatic repulsion between monomers due to its reduced overall charge in comparison with K18 and/or its greater propensity to form β-structure^23^. The 50-fold increase in oligomer population only results in an approximately twofold reduction in the timescale of fibril formation, suggesting an additional effect of the mutation of the transition from oligomers to fibrils. Accordingly, the activation free energy for fibril formation from oligomers also increases on mutation.

Similar results were also obtained for the P301L mutant, with nucleation (and denucleation) rates being increased, but fibril elongation (and shortening) rates being decreased compared with wild type (Table 1). Although appropriate for early times, the fit for this mutant does not describe the later stages of the aggregation process so successfully, indicating the possible presence of additional processes not included in our simplified scheme. However, it remains useful for comparative purposes, so has been used to make approximate quantitative predictions alongside the other proteins studied. Although we are not able to extract reliable estimates for all rate constants for K18-PP, we can make a robust estimate of the change in the nucleation rate on mutation and this is significantly reduced (Table 1).

## Discussion

Interestingly, the two mutants with increased β-sheet-forming propensity display increased nucleation rates, but reduced fibril elongation (and shortening) rates, while the mutant with decreased β-sheet-forming propensity (K18-PP) has a reduced nucleation rate. Although we observed no apparent toxicity (by MTT (3-(4,5-dimethylthiazol-2-yl)-2,5-diphenyltetrazolium bromide) or lactate dehydrogenase assays of K18 oligomers on incubation with neuronal cells in our model system (Supplementary Fig. 6) it is possible that these standard assays are too coarse to detect an effect. A recent study has shown tau oligomers to have more subtle effect by decreasing dendritic spine density of differentiated neurons^29^. We note that the nucleation rates we observed appear to correlate with aggregation of tau within the brain in inducible transgenic mice systems^30^,^31^.

There are several studies based on ensemble methodologies, such as light scattering or ThT fluorescence, of the effect of mutation in tau on aggregation kinetics. These studies are fundamentally quite different to that presented here because these methods being (broadly) sensitive to fibril concentration best report on the later stages of aggregation, rather than the earlier stages addressed here. Nonetheless, we have found that many of our results recapitulate these earlier findings. For example, it has been suggested that the ΔK280 mutation leads to an increased nucleation rate and an enhanced overall aggregation kinetics^10^. Similarly, consistent with the literature, we did not observe any decrease in the concentration of soluble K18-PP tau throughout our measurements. We were, however, able to observe some oligomers, whose number increased during our monitoring period, but still remained lower (1/5th–1/10th) than for the wild-type protein. In general, it is agreed that P301L aggregates faster than wild-type tau^20^,^32^ and it has previously been suggested for full-length tau that this is due to faster fibril elongation^33^,^34^. Under the conditions used here, we observed that despite the rapid formation of oligomers, the reaction approached steady-state more slowly on P301L mutation due to a reduction in the fibril elongation rate constant. There are differing reports of the effect on nucleation, all based on full-length tau, with suggestions that the mutation enhances^32^,^33^, or retards^34^ the process. Our technique, highly sensitive to small oligomeric species, has demonstrated that the nucleation rate is in fact increased due to the mutation, at least in the context of the K18 repeat construct.

It is well known that ‘seeding', the addition of preformed fibrillar or oligomeric structures, generally increases the rate of aggregation in a number of systems, and this has been observed *in vitro* for tau^26^,^35^,^36^. However, experimentally quantifying this effect is challenging due to the difficulty in determining the concentration of these initial seeds. These complications are particularly prominent for oligomeric seeding due to added difficulties in detecting oligomer populations in general. Theoretical modelling thus provides a powerful alternative approach, allowing order-of-magnitude estimates for system properties that are experimentally inaccessible, via careful extrapolation of observations. On the basis of the general conversion model and fit parameters described in Fig. 4, we can estimate the concentration of oligomeric seeds required to double the rate of fibril formation under the experimental conditions described. By following the procedure described in Supplementary Material, we arrive at estimates of 0.01 μM for K18, 0.3 μM for K18-ΔK280 and 0.2 μM for K18-P301L. The difference in values reflects the extent to which the initial nucleation step limits the overall aggregation reaction for each tau construct; for K18, it represents an important bottleneck in the flow of mass from free monomer to fibrils, thus a relatively small contribution to this step via seeding has a larger overall effect. Similarly, we are able to estimate the effects of seeding under conditions of compartmentalization, one of a myriad of complicating factors that occur in the cellular environment. For a typical neuronal cell volume of 6,000 μm^3^ at a tau concentration of 2 μM, the reaction half-time for K18 can be halved from an estimated 4 to 2 h by extremely low numbers of oligomers; ∼30 oligomers suffice, under the assumption that the nucleation reaction is of second order in monomer such that *n*_c_=2. This indicates that only a very small number of seeds would need to be transferred from cell to cell for spreading of oligomers via a prion-like mechanism^37^,^38^, in an idealized situation without inhibition by cellular chaperones. For K18-ΔK280 and P301L under the same conditions, ∼2,000 oligomers are required to approximately halve the reaction half-time from 4 to 2 h and 300 to 150 h respectively. Spreading is thus potentially less efficient for K18-ΔK280, although a 100-fold higher concentration of oligomers, and a fourfold higher mass of fibrils, could be present in the cell as compared with K18, increasing the probability of cellular damage and death. Although these calculations are based on a shortened construct of tau and therefore miss much of the complexity of the biological situation, these calculations may be instructive in understanding the dynamics of spreading. These results are particularly compelling in light of recent results demonstrating differential spreading of tau isoforms between neurons *in vivo*^21^. Following injection of lentiviral particles encoding tau, pathology development for wild-type tau was spread throughout neurons connected to the injection site, whereas pathology associated with P301L tau was restricted to cells close to the injection site despite the higher level of neuronal death observed in the latter case^21^.

In summary, we have combined single-molecule fluorescence and kinetic analysis to determine the aggregation pathway for the tetra-repeat K18 tau construct and two disease-related mutants, allowing us to determine how the mutation alters the aggregation pathway. Our quantitative kinetic model with microscopic rate constants allows us to show that seeding may be highly effective in the cellular environment, providing support for the prion-like spreading model. This could serve as a general approach to determine the critical molecular events that lead to the formation of potentially toxic oligomers, and additionally enables the number of oligomers and aggregation rate to be estimated under conditions that are not accessible experimentally.

## Methods

### Chemicals

Thioflavin T, ammonium acetate and bovine serum albumin (BSA) were all purchased from Sigma. AlexaFluor-488 C5 and 647 C2 maleimide and Vybrant MTT Cell Proliferation Assay Kit are produced by Molecular Probes. Heparin 3,000 was obtained from MP Biomedicals.

### K18 and mutant K18 constructs

To ensure specific labelling, the two natural cysteine residues at positions 291 and 322 (in R2 and R3) were mutated to alanine, and a cysteine was introduced at position 260. This enabled fluorophore labelling of the protein without introducing bulky residues in the repeat domain responsible for aggregation. The construct (K18-C291A/C322A/I260C) was expressed in *E. coli* as described previously^6^, and labelled with either AlexaFluor-488 or AlexaFluor-647 using maleimide chemistry. Mass spectroscopy, SDS–polyacrylamide gel electrophoresis (SDS–PAGE) and analytical size exclusion all showed the protein labelling to have been successful, and the material to be apparently free of preformed aggregates (Supplementary Fig. 1). The aggregation rate of A647-K18 and A647-K18-ΔK280 were similar to that of unlabelled K18 and K18-ΔK280 in the presence of dithiothreitol (Supplementary Fig. 1). Described mutations were achieved through site-directed mutagenesis.

### Aggregation of tau constructs

We employed the same standard aggregation conditions in all our assays to ensure comparability. Solutions of 10 μM K18 construct in 50 mM pH 7.0 ammonium acetate were incubated undisturbed at 37 °C, in the presence of a 1:4 molar ratio of heparin (MW 3000) as an initiator.

### Thioflavin T measurements of tau aggregation

K18 construct was incubated under standard aggregation conditions in the presence of 20 μM Thioflavin T and 5 mM dithiothreitol in untreated half-area 96-well plates (Corning). The fluorescence intensity throughout incubation was monitored using a FLUOstar fluorescence platereader (BMG Labtech, Offenburg, Germany).

### Soluble tau concentrations

In aggregation studies, soluble protein concentrations were monitored by periodic removal of aliquots, and 400-fold dilution, followed by SDS–PAGE analysis. Fluorescence imaging of gels (using a Typhoon Trio, GE Healthcare) was used to quantify band intensity. Concentrations were calculated by comparison with the band intensity of the starting mixture, which was known to be 5 μM A488-K18 and 5 μM A647-K18. The final concentration of soluble tau (roughly 5 μM) also matched that found for the unlabeled construct from SDS–PAGE analysis and Coomassie staining (Fig. 2b, dotted line). In aggregation studies of K18-ΔK280, K18-P301L and K18-PP and disaggregation of K18, the burst rate in the donor channel (using an event threshold of 10) from single-molecule measurements was used as a probe of the concentration of soluble A488-K18. This approach was validated by comparison with results from quantitative SDS–PAGE analysis.

### Single-molecule FRET measurements of K18 aggregation

Aliquots of aggregating mixtures of equimolar A488-K18 and A647-K18 were removed periodically without disturbing the pellet and diluted 50,000-fold into 50 mM pH 7.0 ammonium acetate buffer containing 0.1 mg ml^−1^ BSA and 2.5 μM heparin (average MW 3,000). After dilution, the solutions were loaded into a gel-loading tip attached to the inlet of a simple one-channelled PDMS microfluidic device measuring 25 μm in height and 100 μm in width, as described previously^22^. Sample was passed through the channel by withdrawing solution from the device at the outlet channel at a flow rate of 0.5 cm s^−1^ (achieved by attaching the outlet to a syringe pump (PHD2000, Harvard Apparatus)). The device was mounted onto a home-built single-molecule confocal instrument, described in detail previously^39^. In brief, 488-nm laser light at an intensity of 2 mW was directed through the back port of an inverted microscope (Nikon Eclipse TI) where it was reflected by a dichroic mirror (FF500/646-Di01) through an oil immersion objective (Nikon CFI Plan Apochromat VC 60X Oil N2 NA 1.4, W.D 0.13 mm), which focuses it to a diffraction limited confocal spot within the microfluidic channel. The emitted fluorescence is collected by the same objective and passes through the dichroic mirror, before being focused by a tube lens within the microscope body through a 50 μm pinhole (Thorlabs). A dichroic mirror (585DRLP Horiba) then separates the fluorescence from the two different fluorophores; the longer wavelength passes through the dichroic and is focused by a lens (Plano apo convex, focal length=50 mm, Thorlabs) through a band-pass and long-pass filter 565ALP/695AF55 (both Horiba) onto the Avalanche Photodiode (APD) detector. The shorter wavelength is reflected by the dichroic and is focused through a long-pass and band-pass filter 535AF45 (Horiba)/540LP (Omega optics) onto the second APD. Outputs from the two APDs are connected to a custom-programmed field-programmable gate array, FPGA (Colexica), which counts the signals and combines them into time bins of 100 μs, which matches the expected residence time of the molecules in the confocal volume.

### Analysis of single-molecule FRET aggregation measurements

The time-binned data were analysed using custom-written software in Igor Pro (Wavemetrics), which corrects for both autofluorescence and cross-talk.

The total number of events (monomeric and oligomeric) due to donor (A488)-labelled tau was determined by counting the number of bursts that exceeded 10 counts per bin in the donor channel and used to determine the concentration of soluble donor-labelled tau (by comparison with a solution of known tau concentration, that is, immediately after initiation of the reaction). This approach was validated by cross-checking against SDS–PAGE gels of pelleted samples (see soluble tau concentrations, Methods).

Oligomeric events were then identified as bursts that exceeded 10 counts per bin in the acceptor channel since monomeric tau labelled with either donor or acceptor (A647) would not appear in this channel. We performed experiments to assess the stability of the oligomers during our measurement procedure. ‘Oligomer-rich' samples were generated by incubating K18-ΔK280 for 30 min using our standard conditions and then diluted for (20 min) single-molecule fluorescence measurements. Under these conditions, the oligomer concentration is high enough to examine the oligomer burst rate throughout the measurement. Oligomer concentrations were found to gradually decrease throughout the measurement time within buffers containing BSA (used in our dilution buffer to prevent adsorption of labelled tau to surfaces); however, this affects the overall estimated concentration by less than a factor of two (Supplementary Fig. 5) and does not affect our estimates of the changes in activation energies. It is possible that unstable oligomers, which dissociate within the dead-time of our experiment (a few minutes), are also present in the aggregation reaction mixture; however, such unstable species seem unlikely to impart toxicity and are not required to create a mechanistic model that describes the data. Oligomer concentrations (by number, not mass) were estimated by multiplying the soluble concentration of tau (which is predominantly monomeric) by the proportion of detected events attributed to oligomers. A minor fraction of very intense events spanning multiple time bins was observed in a minority of K18 samples at late time points where sedimented fibrils had been disturbed and was ascribed to fibrillar fragments. To focus on soluble oligomers, these events were excluded from the analysis. Sedimentation was observed reproducibly for K18-wt samples a few hours after initiation of aggregation, but not for any of the K18 mutants. To check that oligomers were not being sequestered within an insoluble fraction and disturbing our analysis, we performed a control experiment where K18 was aggregated as previously but under conditions of continuous shaking to avoid sedimentation. Both the equilibrium monomer concentration and the oligomer concentrations were largely unaffected indicating that the oligomers were not being removed from the solution (Supplementary Fig. 9).

For each of the oligomeric events, the apparent size was calculated by combining the intensity remaining in the donor channel with that transferred to the acceptor channel (after application of a correction factor, *γ*, to account for the different quantum yields and detection efficiencies in the two channels) and comparing this with the average intensity of monomeric events observed in the donor channel. We have used this approach previously^18^, the apparent size being calculated using equation (1)


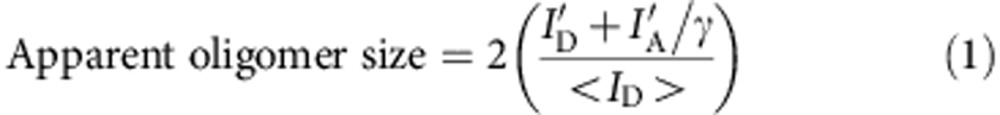


where 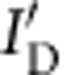 and 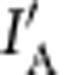 correspond, respectively, to the intensity in the donor and acceptor channels for the oligomeric event, <*I*_D_> represents the average intensity of donor labeled monomers in the donor channel, and the factor of 2 accounts for the fact that on average each oligomer is composed of only 50% donor-labelled tau. Although this last assumption is true on average, it is not true for all oligomers, and this acts to broaden estimated size distributions, particularly for small oligomers. We have previously seen good agreement between size distributions estimated using this approach and for the same oligomers immobilized on surfaces^40^. Following these calculations, events were binned by apparent oligomer size (bin width=1) and FRET efficiency (bin width=0.05), where the FRET efficiency is calculated according to equation (2):


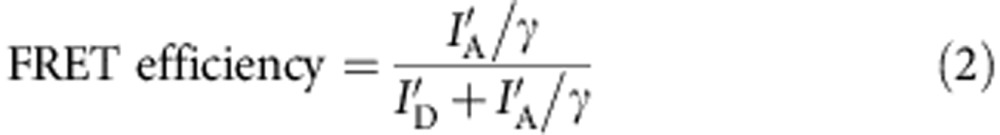


### Single-molecule FRET measurements of filament dissociation

Labelled K18 filaments were prepared using the standard aggregation conditions, and then purified by four cycles of centrifugation where the pellet was washed by replacement of the supernatant with fresh 50 mM pH 7.0 ammonium acetate buffer. Following the final wash, 40–65 μl of fresh buffer was carefully pipetted above the pellet without disrupting it. Aliquots of solution (away from any pellet) were removed periodically, rapidly diluted into 50 mM pH 7.0 ammonium acetate and placed on BSA-treated microscope slides (not assessed under flow conditions). These experiments were performed using a different instrument to the aggregation assays. The set-up, experimental and data analysis protocols have been described in detail previously^18^,^39^, and were subject to only the following minor modifications. The 488-nm laser was used at a power of 80–90 μW. We identified bursts above a threshold of 7 photons ms^−1^ bin in the acceptor channel as being due to oligomers. In support of this approach, buffer-only controls analysed in this manner typically resulted in only ∼1 % of the number of events as those detected in dissociation reactions.

### Cell culture thiazolyl blue tetrazolium bromide (MTT) assay

SH-SY5Y cells (from the European Collection of Cell Cultures) were cultured at 30 × 10^3^ cells per well in a 96-well plate in DMEM supplemented with 10% (v/v) fetal calf serum, 1% (v/v) penicillin/streptomycin in a 5% CO_2_ environment at 37 °C for 24 h. After 24 h, the medium was replaced with 50 μl of fresh culture medium. Samples of K18-ΔK280 were aggregated using the standard conditions to result in maximal concentration of oligomers (0.5 h for K18-ΔK280). Dilutions of K18-ΔK280 were made up in DMEM and then 50 μl of sample was added to each well. A positive control of 5 μM Staurosporin and negative controls of HBSS and medium alone were also added to the cells. The cells were left to incubate for 4 h in a 5% CO_2_ environment at 37 °C. The media were then removed and 100 μl of fresh medium was added to each well. Ten μl of 12 mM MTT stock solution was then to each well and incubated at 37 °C for 2 h. All but 25 μl of medium was then removed from the cells and 50 μl of dimethylsulphoxide was added to each well and mixed thoroughly with a pipette. The cells were then incubated for 10 min at 37 °C. The plates were shaken for 1 min and absorbance at 540 nm for each sample determined with a FLUOstar Optima plate reader (BMG Labtech). Absorbance values of SH-SY5Y cells in different experimental conditions were normalized against cells treated with DMEM media.

### Failure of classical nucleated polymerization theory

The classical theory of nucleated polymerization^27^,^28^ provides a simple yet extraordinarily powerful description of aggregating filamentous systems. With only minor modifications, this framework has led to successful descriptions for the kinetics of a wide range of proteins^16^,^27^,^28^,^41^,^42^. A notable simplification that renders these models so useful is the identification of only two species in the reaction pathway: monomers and fibrils. Any oligomeric species are treated simply as small fibrils, and off-pathway reactions are neglected.

The master equation describing the kinetics of a nucleation–polymerization reaction is illustrated in Fig. 5, and reads


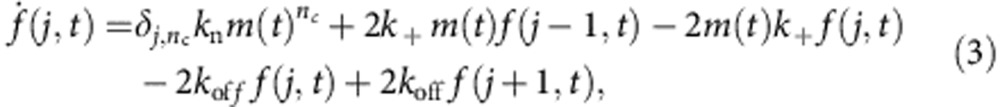


where *δ*_*x,y*_ denotes the Kronecker symbol. The critical nucleus size is denoted *n*_c_, with a corresponding nucleation rate constant *k*_n_; *n*_c_ represents the number of monomer residues that make up the smallest stable aggregate. Although heparin plays an important catalytic role in the nucleation reaction, its effects are included in the nucleation rate constant. The symbol 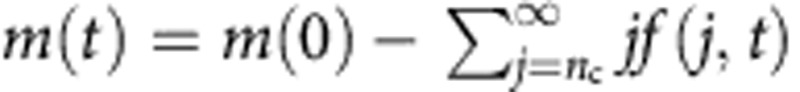 denotes the concentration of free monomer as a function of time, with *m*(0) denoting the initial monomer concentration; 10 μM for all experiments here. The concentration of fibrils containing *j* monomeric residues is denoted *f*(*j, t*), with *f*(*j*, 0)=0 for all *j*≥*n*_c_ and *f*(*j, t*)=0 for all *j*<*n*_c_. Fibril elongation is described by the rate constant *k*_+_, with dissociation of monomer from fibril ends governed by *k*_off_; the factor of 2 allows for two free ends per fibril. Both elongation and dissociation rate constants are thus assumed independent of fibril length.

With careful treatment of boundary terms^16^,^17^, equation (3) can be summed to yield an expression for the zeroth moment of the fibril length distribution, 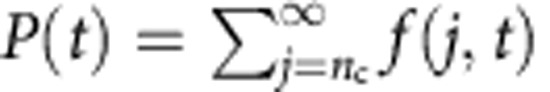:


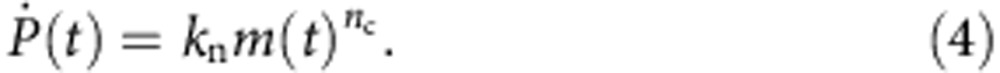


We can formally integrate equation (4) to give


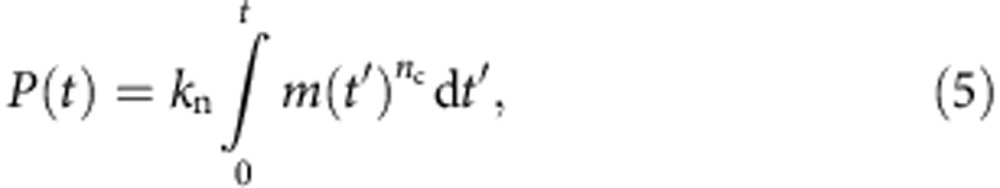


representing the total concentration of aggregates as a function of time.

Supplementary Fig. 8b–d illustrates how equation (5) can be used to obtain an upper bound for the average aggregate length distribution within this framework that is consistent with the kinetic available data for each K18 tau construct. For each data set, the time series for *m*(*t*) can be inferred experimentally from measurements of the total concentration of soluble tau species, accounting for the known proportion of which is accounted for by oligomers (see Figs 3 and 4). The resulting numerical function is linearly interpolated (Supplementary Fig. 8b) and integrated to find *P*(*t*) as per equation (5), with *k*_n_ set to the smallest possible value that ensures *P*(*t*) accounts for observed oligomer populations (Supplementary Fig. 8c). This lower bound for *P*(*t*) is then used to obtain an upper bound for the average aggregate length μ(*t*) as a function of time (Supplementary Fig. 8d), via the relation


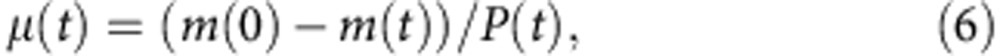


where *m*(*0*)–*m*(*t*) gives the total concentration of monomeric residues contained within aggregates.

By applying this procedure for a range of plausible critical nucleus sizes (2≤*n*_c_≤10), we show that the maximum plausible average aggregate length allowed by classical nucleation–polymerization theory falls short of observed tau fibril lengths formed under these conditions^23^. In addition, the *n*_c_=10 case more generally demonstrates the failure of any model where monomers only form a single type of aggregate, provided that the total aggregate concentration does not decline beyond the time at which the observed oligomer population reaches a peak. Furthermore, by assuming a reasonable average fibril length of 2,000 monomer units throughout the reaction (a 1-μm fibril with one tau molecule in each β-strand), the aggregate concentration *P*(*t*) can be found numerically from equation (6), using the experimental time series for *m*(*t*). These *P*(*t*) curves thus represent strong upper bounds for the oligomer concentration *x*(*t*), as shown overlaid in dotted black in Fig. 4b. Although at early reaction times this upper bound is underestimated due to transient smaller initial average aggregate size, at later times it is clear that classical nucleation–polymerization does not provide an adequate description. A modification to this basic theory is thus needed to reconcile observed oligomer concentrations with the production of long fibrils.

### Conversion model and fitting

Oligomeric aggregates are now treated as distinct from fibrils, introducing an additional step in the reaction pathway: oligomers now exchange both with free monomer via a reaction of order *n*_c_ in the monomer concentration, and with fibrils via a reaction of first order in monomer concentration. Supplementary Fig. 10 and Fig. 4a illustrate this new model, demonstrating how it is coarse-grained, simplified and re-expressed in a useable form in terms of conversions between different species with established average sizes. The kinetic equations for this simple model read:


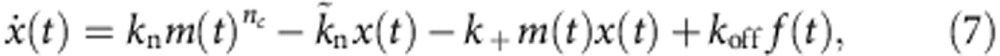



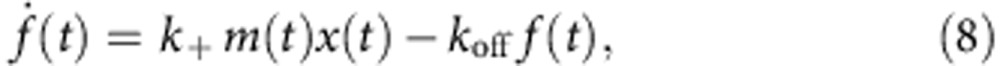



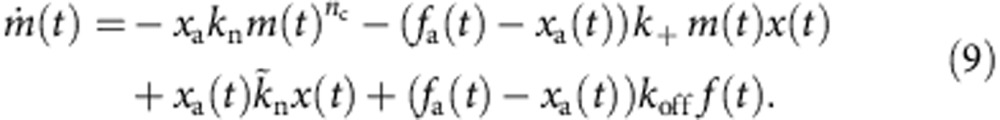


Concentrations of different populations are denoted with the symbols

*x*(*t*): oligomeric species,

*f*(*t*): fibrils,

*m*(*t*): monomers,

where the initial values *x*(0) and *f*(0) are 0, and *m*(0)=10 μM as before. Heparin dependence is again subsumed into the nucleation rate constant. The critical nucleus size *n*_c_ is now taken as *n*_c_=2 for simplicity.

The various average lengths of different species, expressed as numbers of monomeric residues, are given by:

*x*_*a*_(*t*): average length of oligomers,

*f*_*a*_(*t*): average length of fibrils

and the various rate constants describe the processes shown in Fig. 4a and Supplementary Fig. 10b:

*k*_n_: heparin-dependent nucleation of monomers to form oligomers,


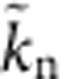
: complete dissociation of oligomers into monomers,

*k*_+_: monomer-dependent conversion of oligomers to fibrils,

*k*_off_: conversion of fibrils back to small oligomers, releasing free monomer.

To conserve mass, the average lengths *x*_*a*_(*t*) and *f*_*a*_(*t*) must be constant with time. The oligomer length, averaged over the full reaction time, can be determined directly from the available data giving numbers of oligomers detected of each length. Average fibril length is estimated at 2,000 monomeric residues per fibril, although the quality of fit is not strongly sensitive to the precise value chosen.

The functions *m*(*t*) and *x*(*t*) are fitted directly to populations inferred from the data presented in Figs 3 and 4, via numerical integration of the kinetic equations. All fits are numerical, with four rate constants fitted separately to each tau data set using a global least-squares Levenberg–Marquardt algorithm. The resulting best fits are shown in Fig. 4b.

### Seeding predictions

The effects of seeding are taken into account straightforwardly, by setting the initial population concentrations in equations (7, 8, 9) at *t*=0 according to the nature of the seeds. All rate constants are set according to the fitting procedure discussed above. For the simulations described in the main text, we investigate seeding by oligomers; *x*(0) is set to the initial seed concentration, while *f*(0) remains negligible. The rate of aggregation is monitored via the formation of fibril mass; if the unseeded reaction gives a final fibril residue concentration *f*_*a*_·*f*(∞)=*W*, and at time *t*_*W*/2_ has produced a fibril residue concentration of *W*/2, any increase in reaction rate on seeding is quantified by considering the time ^*s*^*t*_*W*/2_ at which the seeded reaction produces a fibril residue concentration of *W*/2. The seeding required to double the reaction rate is thus found by setting ^*s*^*t*_*W*/2_/*t*_*W*/2_=1/2. For the K18 wild-type construct, this criterion is very similar to simply halving the reaction half-time. For the ΔK280 deletion mutant, seeding leads to a non-negligible increase in the total fibril mass produced, thus motivating this more careful definition. Intracellular conditions are simulated by setting *m*(*0*)=2 μM.

### Inferring activation free energies

For a given rate constant *k* describing a particular reaction step, the activation free energy Δ*G*^‡^ of the process at a temperature *T* can be inferred from transition state theory^44^, via the equation


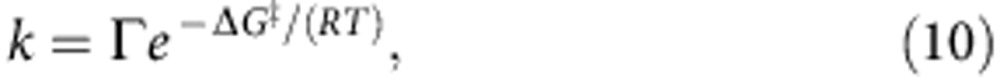


where *R* represents the molar gas constant. The prefactor Γ has units matching those of *k*, and is approximately constant for a given process; it has a weak temperature dependence that to a good approximation is assumed negligible in the studied range. The change in activation energy ΔΔ*G*^‡^ for a given process on mutation can thus be found^44^ independently of Γ by applying equation (10) to both the WT (*k*_WT_) and the Mutant (*k*_Mut_) system and subtracting:


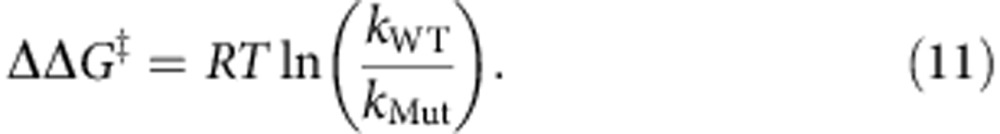


The values quoted in Table 1 are thus found by application of equation (11) to the four different rate constants in the conversion model.

## Author contributions

S.L.S. designed and performed the single-molecule fluorescence experiments, bulk kinetic assays, size-exclusion chromatography, SDS–PAGE and cell toxicity studies. S.L.S. also analysed the data and wrote the paper. G.A.G. developed the kinetic model and wrote the paper. S.K. designed and performed the electron microscopy studies. N.S. designed and performed the cell toxicity studies. M.K. designed and performed the single-molecule fluorescence studies. M.H.H. designed the single-molecule fluorescence studies. D.K., T.P.J.K and E.M supervised the project and edited the manuscript.

## Additional information

**How to cite this article:** Shammas, S.L. *et al.* A mechanistic model of tau amyloid aggregation based on direct observation of oligomers. *Nat. Commun.* 6:7025 doi: 10.1038/ncomms8025 (2015).

## Supplementary Material

Supplementary Information Supplementary Figures 1-10 and Supplementary Table 1

## Figures and Tables

**Table 1:** The effect of mutation on the four different activation free energies according to a simple oligomeric conversion model for tau aggregation.

**Protein**	***k***_***n***_	***ǩ***_***n***_	***k***_***+***_	***k***_**off**_
ΔK280	−4.9±0.7	−1.5±0.7	+2.5±0.6	+1.7±1.0
P301L	−2.7±0.7	−1.8±0.7	+3.1±0.8	+4.6±1.1
PP	4.1±0.7	ND	ND	ND

ND, not determined; PP, I277P I308P mutant.

The change in activation energies (from K18-wt) for the forward and backward processes of nucleation to form oligomers from monomer (*k*_n_ and *ǩ*_n_) and the conversion of oligomers into fibrils (*k*_*+*_ and *k*_off_) are reported in units of RT for each mutant.
