# Supplementary material for: A mechanistic model of tau amyloid aggregation based on direct observation of oligomers
Source: Nat Commun. 2015 Apr 30;6:7025. doi: 10.1038/ncomms8025 (PMC4421837; doi:10.1038/ncomms8025)
Supplement: Supplementary Information — Supplementary Figures 1-10 and Supplementary Table 1 [file ncomms8025-s1.pdf]

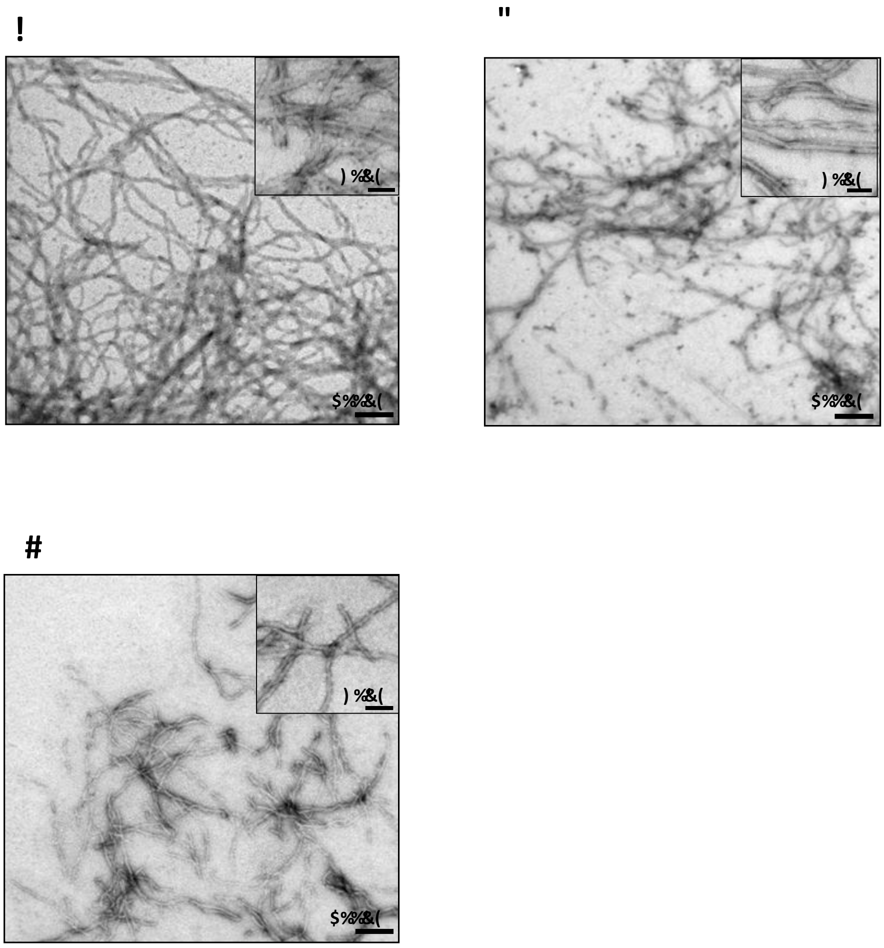

Supplementary Fig. 1. A488-K18 and A647- K18 constructs aggregate into filamentous amyloid fibrils. TEM images show paired helical filaments are present after 6 hours when K18 (A), A488-K18 (B) or A647-K18 (C) are incubated under the standard aggregation conditions.

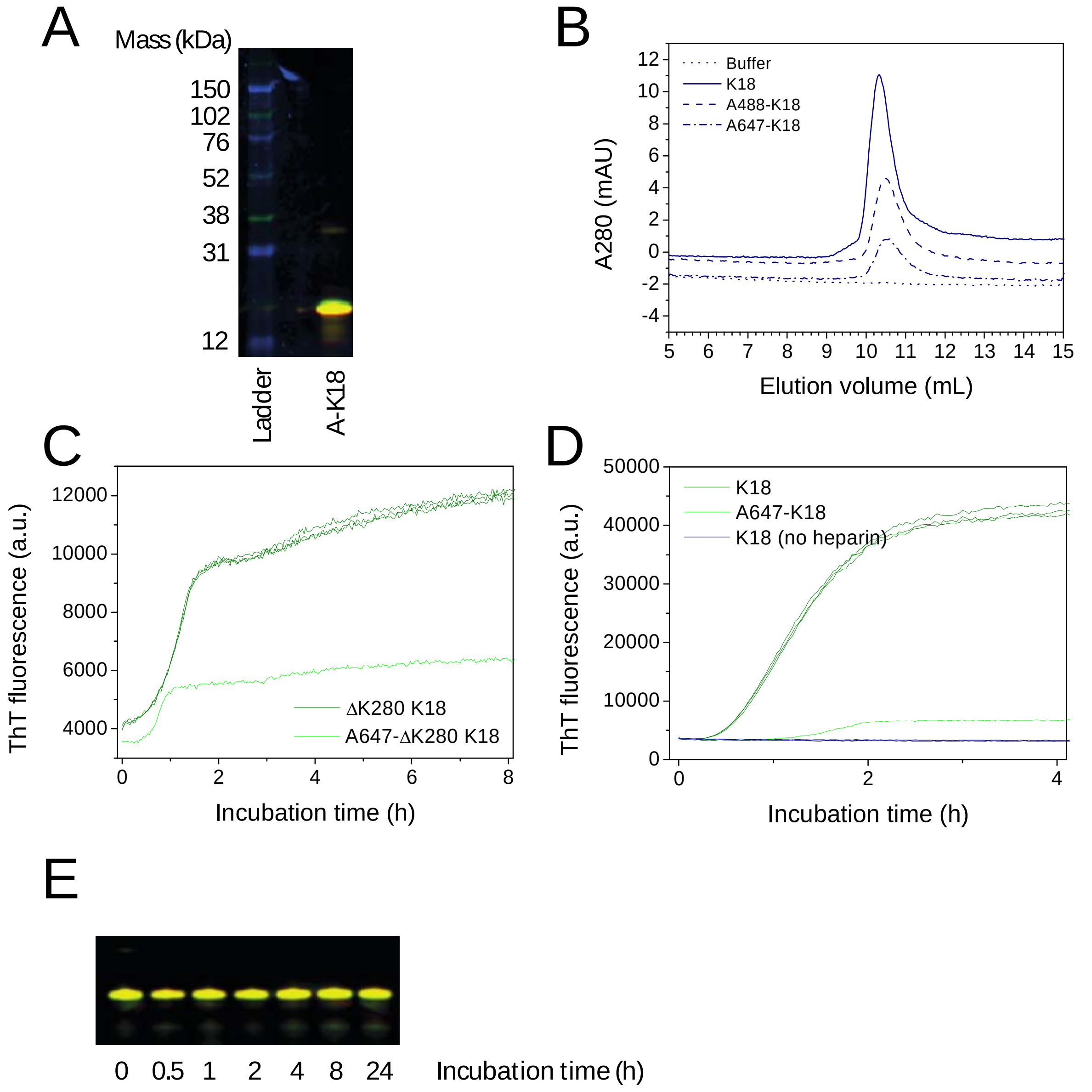

Supplementary Fig. 2. Characterization of A488-K18 and A647-K18 by ensemble methods (A) SDS-PAGE of a 1:1 mixture immediately following heparin addition, visualized by fluorescence. The material eluted at a similar apparent mass as unlabeled construct. K18 constructs are resistant to degradation under standard experimental conditions for over 24 hours (E). (B) Analytical SEC on a Superdex 30/100 column showed the solutions were free of large quantities of pre-aggregated material (void of column found to be 7.7 ml) and that the labeled constructs were of a similar size to the unlabeled construct. Globular standards suggest the mass is around 50 kDa, which would be 3-4 K18 monomers, however the tau protein is known to be predominantly disordered and disordered proteins are know have shorter retention times in SEC than folded proteins of similar size. (C) and (D) Aggregation kinetics in the absence and presence of an Alexa-647 label for K18 (C) and K18-ΔK280 (D) respectively, measured by ThT fluorescence. All samples were in triplicate except A647-K18.

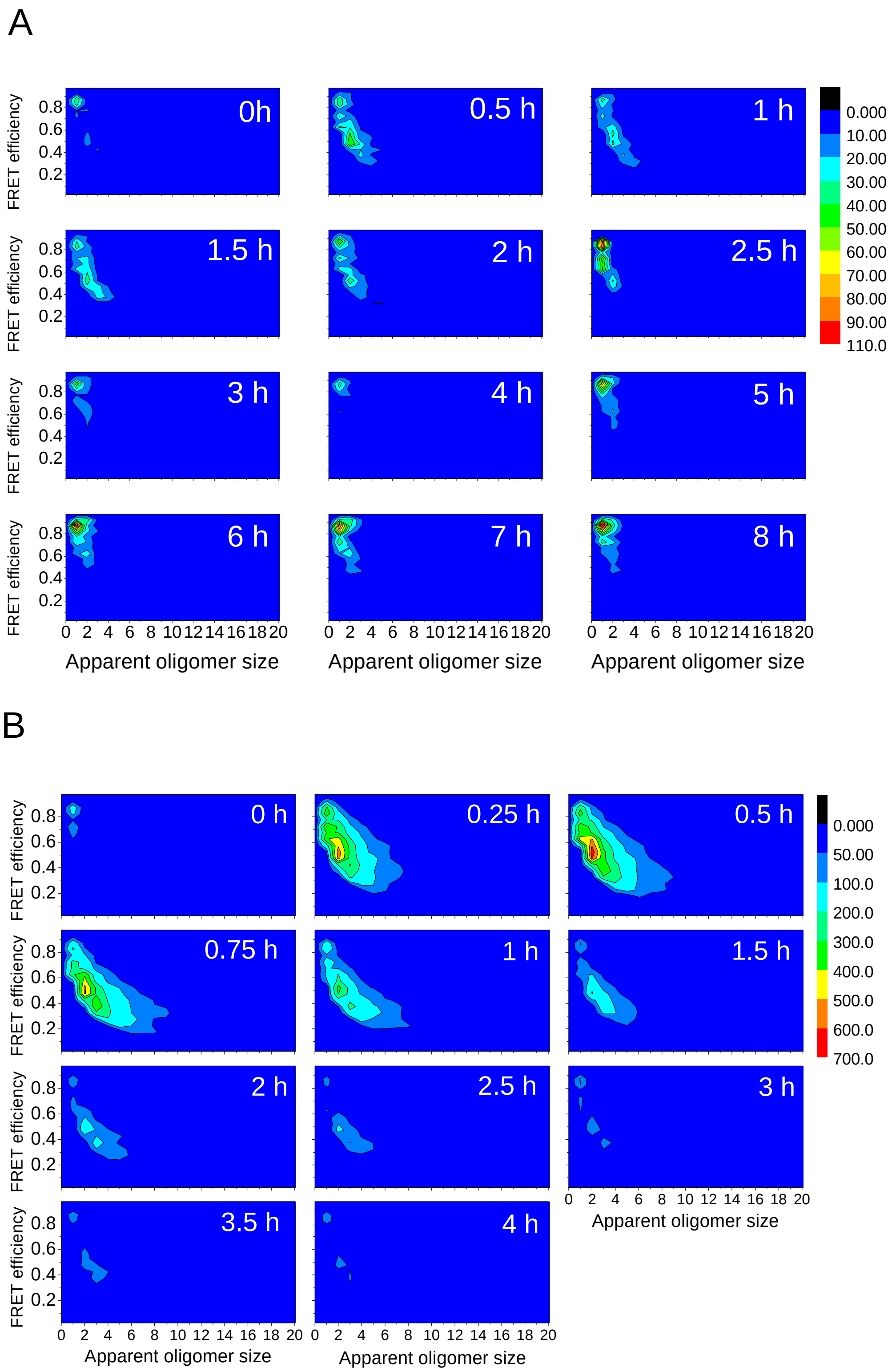

Supplementary Fig. 3. FRET-apparent size contour plots (by number) for observed oligomers during aggregation of (a) A488/A647-K18 and (b) A488/A647- K18-ΔK280. At intermediate timepoints slightly larger medium-FRET efficiency oligomers are observed, however oligomers are small throughout.

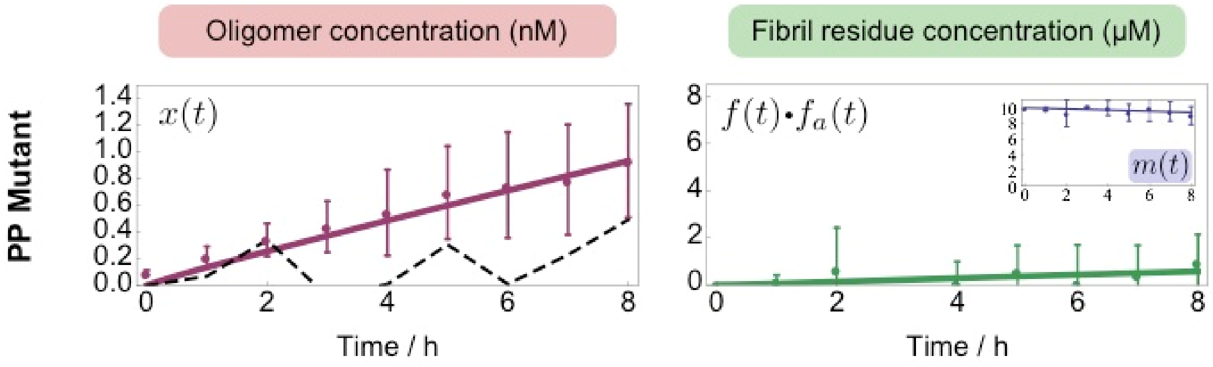

Supplementary Fig. 4. Oligomeric and soluble (monomeric) concentrations observed during incubation of A488/A647-K18-PP our standard aggregation conditions, and the correspondingly deduced fibril concentration. Oligomeric concentrations are significantly lower than those observed for K18-wt, and increase throughout the incubation without an observable decrease in soluble tau concentrations (production of filaments).

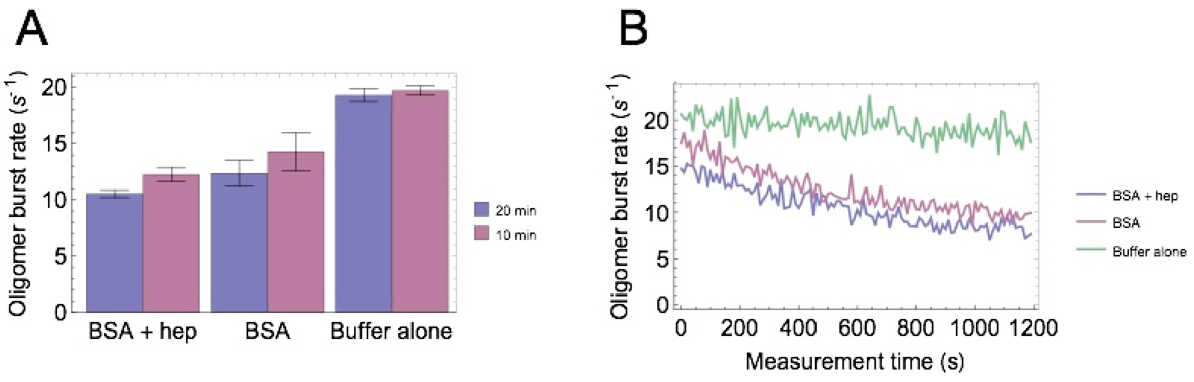

Supplementary Fig. 5. Estimated oligomer concentrations depend upon the average oligomer burst rate throughout the measurement period (10 mins or 20 mins). The dependence of this parameter (A), which relates to the oligomer stability upon dilution for single molecule measurements, on the dilution buffer was investigated by monitoring the oligomer burst rate throughout the measurement time (B) using a sample of K18-ΔK280 incubated under our standard conditions for 30 minutes (to maximize the number of oligomers). The results in this figure are the average of three replicate experiments.

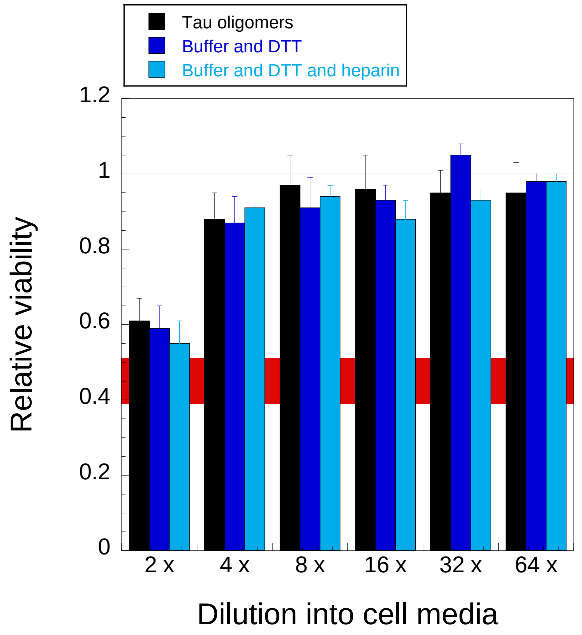

Supplementary Fig. 6. Tau oligomers are not toxic (when applied externally). Unlabeled K18-ΔK280 was applied externally to SH-SY5Y cells (human neuroblastoma cells) for four hours and cell viability assessed by an MTT assay. Cells were exposed to tau construct pre-incubated under standard aggregation conditions in the presence of DTT for 0.5 hours (black bars). This timescale was chosen to maximize the oligomeric concentration. Samples of tau were not more toxic than matched buffer controls according to this assay. The red bar represents the viability of cells treated with a positive control (staurosporin) for comparison. Experiments were performed four times in quintuplicate, and sample absorbance, which is taken as a measure of cell viability, is reported relative to the absorbance of cells treated with DMEM media alone.

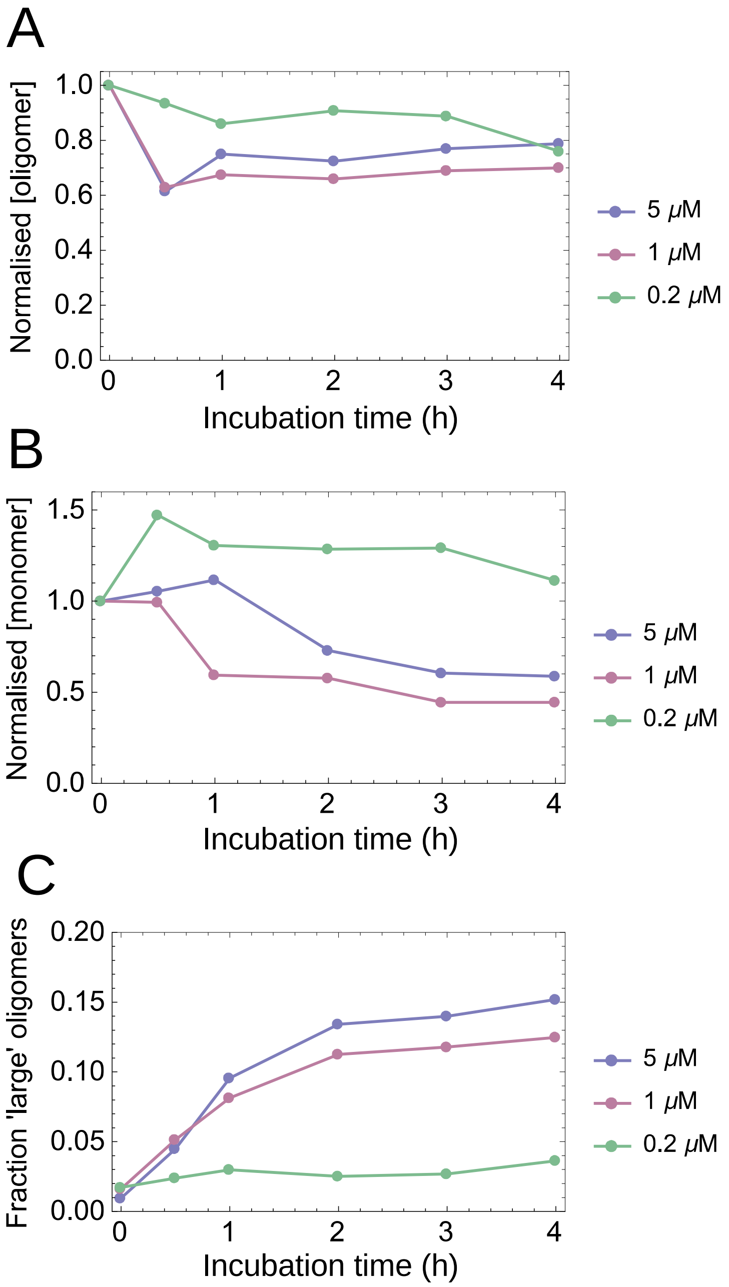

Supplementary Fig. 7. Tau oligomers remain stable in cellular media under cellular assay conditions. Oligomer-rich samples of K18-ΔK280 were generated by incubating them under standard conditions for 30 minutes, and then diluted with cell media to concentrations used in cellular assays and incubated under cellular assay conditions for up to 4 hours (the incubation time used in cellular assays). Burst rates from oligomers (A) and soluble tau (B) are reported relative to the burst rate measured immediately after dilution into media. At the higher concentrations there was some indication that aggregation was continuing in the media after dilution as the fraction of large oligomers increased with incubation time (C).

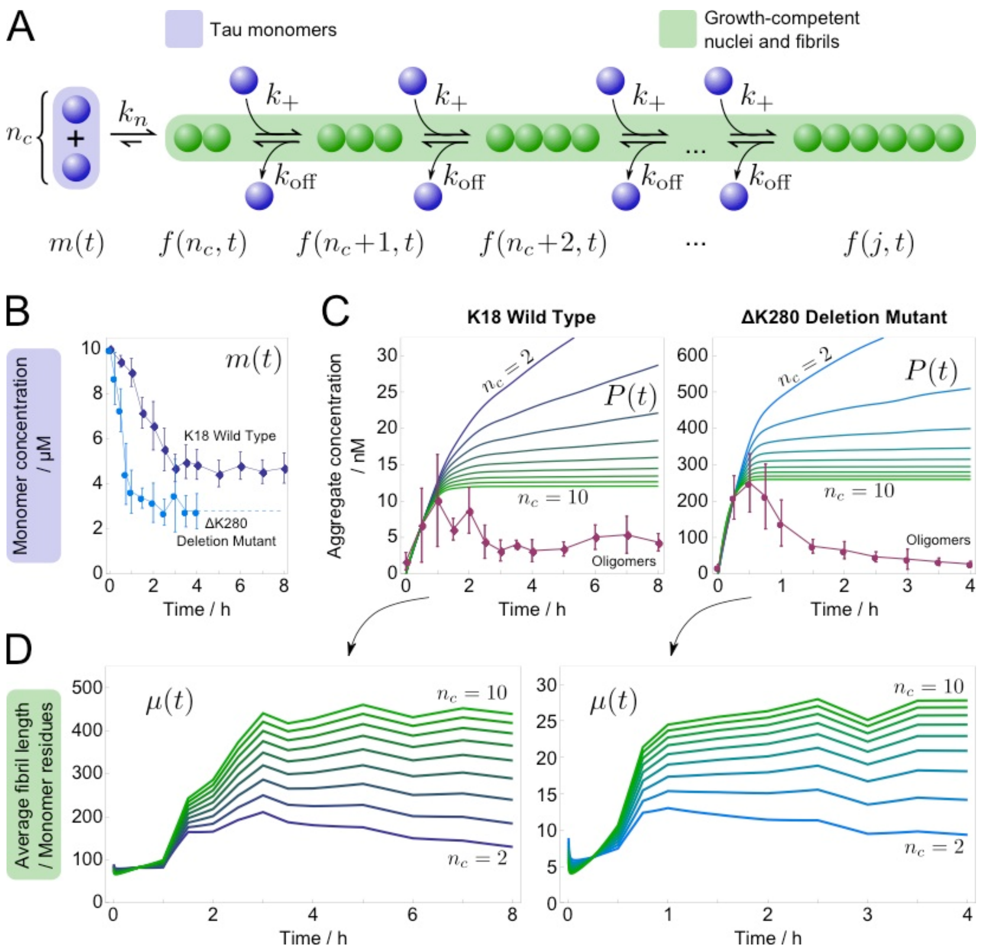

Supplementary Fig. 8: Failure of a simple nucleation-polymerisation model in describing tau aggregation data. (A) Schematic of kinetic model. A nucleation reaction of order n_c in free monomer concentration $m(t)$ gives rise to small aggregates of n_c residues, with concentration $f(n_c, t)$. Successive elongation reactions of first order in monomer concentration allow these aggregates to grow, with dissociation reactions opposing this process. Rate constants are labelled for each process, with elongation and dissociation assumed independent of aggregate size j. Contributions from the reverse reaction for nucleation are assumed negligible. (B) Experimental data for free monomer concentration $m(t)$, for both K18 wild type tau constuct, and its deletion mutant ΔK280. These timecourse can be linearly interpolated as shown. (C) Curves for total aggregate concentration, $P(t) = \sum_{j=n_c}^{\infty} f(j,t)$, can be obtained numerically from $m(t)$ via Eq. 3. Resulting curves are shown for a realistic integer nucleus size range of $2 \le n_c \le 10$, with the minimum value for k_n chosen such that $P(t)$ can still account for observed oligomer populations, shown overlaid. (D) The resulting lower bounds for $P(t)$ allow estimation of upper bounds for the mean fibril length $\mu(t)$, expressed in numbers of monomer residues, via the relation $\mu(t) = (m(0) - m(t))/P(t)$. Even for the relatively large critical nucleus size of $n_c = 10$, the mean fibril length consistent with observations, according to a nucleation-polymerisation model, falls short of the approximately 2000 monomers expected in tau fibrils (see Materials and Methods). This description thus cannot account for experimental observations.

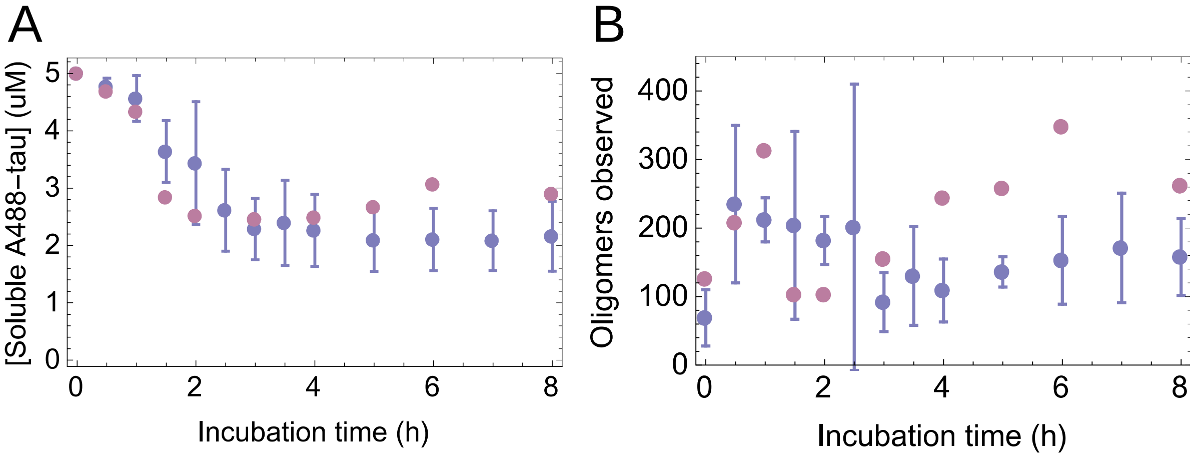

Supplementary Fig. 9. Similar soluble tau concentrations (A) and oligomer concentrations (B) are observed during the aggregation of A488/A647-K18 aggregation under quiescent (purple) and agitated (pink) conditions.

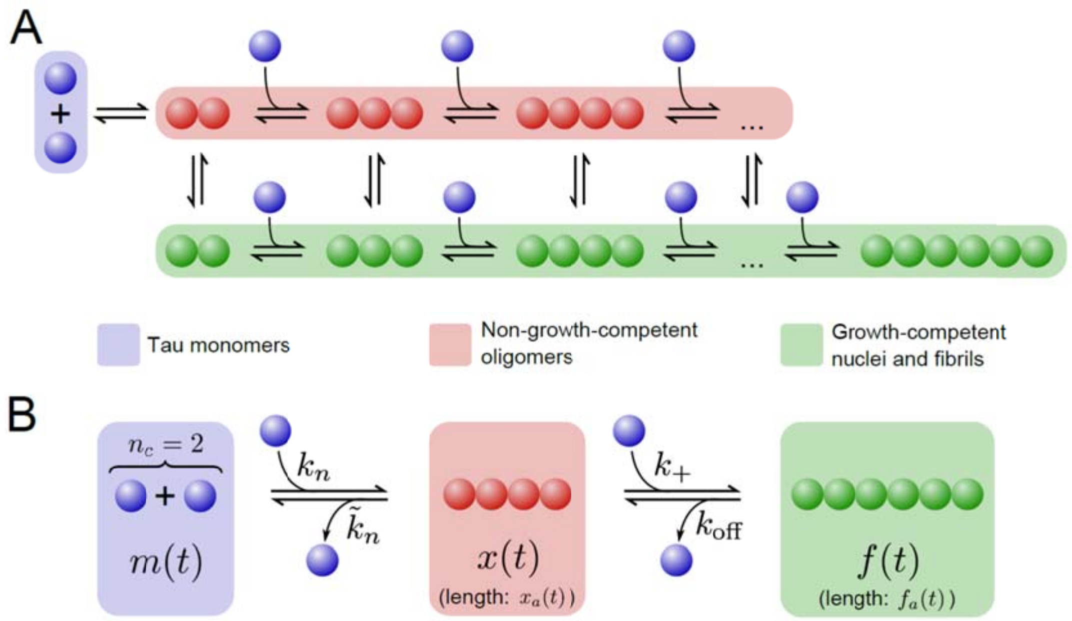

Supplementary Fig. 10: Nucleation-conversion-polymerisation description of tau aggregation. (A) General on-pathway conversion model, with nucleation giving rise to an oligomer population that must undergo a further reaction, such as a structural conversion, before generating growth-competent nuclei. Aggregation occurs via addition of monomer units to these nuclei via successive reactions of first order in the monomer concentration, in competition with dissociation reactions; release of monomers at each dissociation step is omitted for clarity. **(B)** Coarse-grained simplification of conversion model, used to interrogate experimental data as described in the text; Fig. 4 describes this coarse-grained model in more detail.

Protein	k_n / µM^{-1} h^{-1}	$\check{k}_n$ / h^{-1}	k_+ / µM^{-1} h^{-1}	k_{off} / h^{-1}
WT	$(9.3 \pm 3.5) \times 10^{-5}$	$(6.1 \pm 3.0) \times 10^{-1}$	$(3.2 \pm 1.5) \times 10^{-2}$	$(1.9 \pm 1.0) \times 10^{-1}$
ΔK280	$(1.3 \pm 0.7) \times 10^{-2}$	2.7 ± 1.5	$(2.5 \pm 1.1) \times 10^{-3}$	$(3.4 \pm 3.0) \times 10^{-2}$
P301L	$(1.5 \pm 0.8) \times 10^{-3}$	3.7 ± 1.7	$(1.4 \pm 0.9) \times 10^{-3}$	$< (2.0 \pm 2.0) \times 10^{-3}$
PP	$(1.6 \pm 0.9) \times 10^{-6}$			

Supplementary Table 1. Rate constants for the fits presented in Fig. 4 for the various processes according to the coarse-grained model presented in Fig. S10. These values are presented for the interest of the reader. Unlike the changes in activation energies reported upon mutation (Table 1) they are not insensitive to the absolute values of oligomer concentration, and are thus considered less robust. For example if our oligomer concentration is assumed to be a 2-fold underestimate, then this causes roughly 2-fold increases in estimated values of k_n and $\check{k}_n$, and 2-fold decreases in estimated values of k_+ and k_{off}.
